# An E-Liquid Flavor Wheel: A Shared Vocabulary Based on Systematically Reviewing E-Liquid Flavor Classifications in Literature

**DOI:** 10.1093/ntr/nty101

**Published:** 2018-05-18

**Authors:** Erna J Z Krüsemann, Sanne Boesveldt, Kees de Graaf, Reinskje Talhout

**Affiliations:** 1 National Institute for Public Health and the Environment (RIVM), Centre for Health Protection, Antonie van Leeuwenhoeklaan, MA Bilthoven, The Netherlands; 2 Division of Human Nutrition and Health, Wageningen University, Stippeneng, WE Wageningen, The Netherlands

## Abstract

**Introduction:**

E-liquids are available in a high variety of flavors. A systematic classification of e-liquid flavors is necessary to increase comparability of research results. In the food, alcohol, and fragrance industry, flavors are classified using flavor wheels. We systematically reviewed literature on flavors related to electronic cigarette use, to investigate how e-liquid flavors have been classified in research, and propose an e-liquid flavor wheel to classify e-liquids based on marketing descriptions.

**Methods:**

The search was conducted in May 2017 using PubMed and Embase databases. Keywords included terms associated with electronic cigarette, flavors, liking, learning, and wanting in articles. Results were independently screened and reviewed. Flavor categories used in the articles reviewed were extracted.

**Results:**

Searches yielded 386 unique articles of which 28 were included. Forty-three main flavor categories were reported in these articles (eg, tobacco, menthol, mint, fruit, bakery/dessert, alcohol, nuts, spice, candy, coffee/tea, beverages, chocolate, sweet flavors, vanilla, and unflavored). Flavor classifications of e-liquids in literature showed similarities and differences across studies. Our proposed e-liquid flavor wheel contains 13 main categories and 90 subcategories, which summarize flavor categories from literature to find a shared vocabulary. For classification of e-liquids using our flavor wheel, marketing descriptions should be used.

**Conclusions:**

We have proposed a flavor wheel for classification of e-liquids. Further research is needed to test the flavor wheels’ empirical value. Consistently classifying e-liquid flavors using our flavor wheel in research (eg, experimental, marketing, or qualitative studies) minimizes interpretation differences and increases comparability of results.

**Implications:**

We reviewed e-liquid flavors and flavor categories used in research. A large variation in the naming of flavor categories was found and e-liquid flavors were not consistently classified. We developed an e-liquid flavor wheel and provided a guideline for systematic classification of e-liquids based on marketing descriptions. Our flavor wheel summarizes e-liquid flavors and categories used in literature in order to create a shared vocabulary. Applying our flavor wheel in research on e-liquids will improve data interpretation, increase comparability across studies, and support policy makers in developing rules for regulation of e-liquid flavors.

## Introduction

Electronic cigarettes (e-cigarettes) vaporize e-liquids, which consist of a propylene glycol and glycerol base, and a varying amount of nicotine and flavorings.^1^ Flavorings are the flavor molecules present in e-liquids that contribute to the perceived flavor, whereas we refer to flavors as the combined sensations of taste and smell of e-liquids from a particular brand. The number of available e-liquid flavors exceeded 7500 in 2014 and is still increasing.^2^ These flavors increase sensory appeal of the e-liquid.^3^ Increasing attractiveness of e-liquid flavors could stimulate smokers to use an e-cigarette as alternative for regular cigarettes, as nontobacco and nonmenthol flavors are associated with higher rates of smoking cessation.^4–6^ On the other hand, it is well established that flavors in tobacco products generally attract adolescents and youth.^7–10^ Flavor preferences may also play an important role in *e-cigarette* use among adolescents.^11^ Especially nontobacco e-liquid flavors are attractive to nonsmoking youth, thereby stimulating use and nicotine consumption.^12–14^

Nicotine-containing e-liquids have a stimulating effect on the reward system within the brain, which is implicated in the development of addiction.^15^ The core psychological components of reward are liking, learning, and wanting.^16^ Whereas flavors are added to increase product liking, addictive substances such as nicotine play a role in motivation and influence the reward system through mechanisms of learning and wanting. Considering existing literature, research has mostly focused on the role of flavors in liking of e-cigarettes, providing insight in e-cigarette use and preferences. For instance, a review of Huang et al.^17^ showed that most e-cigarette users prefer nontraditional flavors such as fruit and sweet flavors compared to traditional flavors such as tobacco or menthol. In addition, a recent study showed that adolescents predominantly prefer fruit, candy/dessert, and vanilla, whereas the most preferred flavors among adults are nonsweet e-cigarette flavors such as fruit, tobacco, and menthol/mint.^11^ For regulation purposes, it is important to understand how flavor liking differs among different consumer groups, for example, adult tobacco smokers and nonsmoking adolescents or youth. However, as the variety of available e-liquid flavors increases and more and more research is being conducted, a systematic way of flavor classification is needed in order to increase comparability of results and facilitate data interpretation among researchers and policy makers.

Flavor wheels have been developed as a tool to consistently classify flavors and/or aromas in the food, alcohol, and fragrance industries. A flavor wheel visually represents a shared vocabulary of flavor attributes that are classified into general categories. For instance, Noble et al.^18^ developed a wine aroma wheel in 1984 containing 12 main categories such as fruity, vegetative, nutty, earthy, chemical, floral, and spicy, and uses subattributes for specification. Similarly, flavor wheels have also been developed for other alcoholic beverages (eg, beer and whiskey),^19,20^ for food products (eg, chocolate, coffee, olive oil, and cheese),^21–24^ and for fragrances.^25^ Regarding tobacco products, the industry has created a cigar flavor wheel that consists of 8 main categories and 52 subcategories.^26^ These flavor wheels are used as a common vocabulary within industries and science, for instance, as a tool used by consumer or expert panels to assess flavor attributes.

While the number of unique e-cigarette flavors is increasing, no flavor wheel for e-liquids currently exists. We have reviewed e-liquid flavor classification in existing literature and propose a flavor wheel to systematically classify e-liquid flavors.

The importance of developing a systematic flavor classification for e-liquids was previously mentioned by Yingst et al.,^27^ who conducted a survey about participants’ favorite e-liquid flavor. The researchers used the participants’ responses to develop a list of flavor categories and guidelines for classification of e-liquid flavors. Flavor classifications may differ across study disciplines, as individuals interpret e-liquid brand names and marketing descriptions in a different way. We therefore reviewed existing literature (including the publication of Yingst et al.) to investigate which classifications and terminology researchers have used in order to find a commonly agreed flavor vocabulary.

To develop a shared vocabulary, we propose an e-liquid flavor wheel that summarizes flavor categories from literature. The flavor wheel could be applied to multiple research disciplines, for instance, to investigate liking of particular flavor categories among different consumer groups. Applying our flavor wheel for e-liquids will facilitate communication among and between researchers, consumers, and policy makers, which will improve data interpretation and increase comparability of results across studies.

## Methods

### Data Sources and Search

Our search strategy aimed to identify peer-reviewed journal articles in which flavors are investigated in relation to e-cigarette use and preferences. The strategy was developed with the assistance of an experienced librarian with expertise in conducting and documenting literature searches. The search was conducted in May 2017 using PubMed and Embase databases. The search was updated to include current literature up to January 2018. Keywords included terms to capture concepts associated with e-cigarettes, flavors, liking, learning, and wanting. Articles published between the year of 1990 and the search date were included. As an example, the complete search strategy for the PubMed database is added in Supplementary Table 1.

### Study Selection and Exclusion Criteria

**Figure 1.**
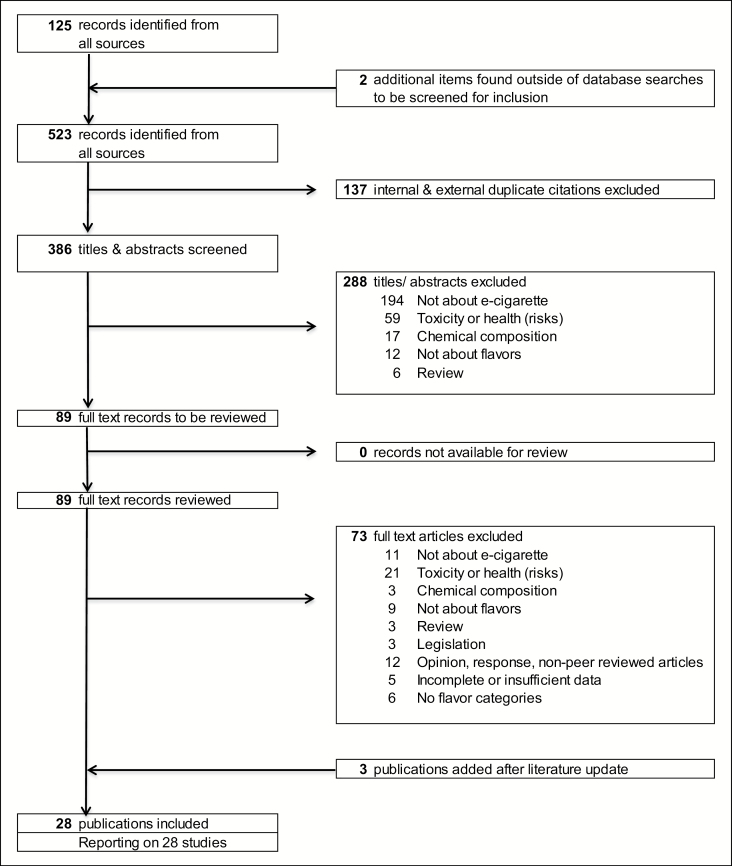
PRISMA flowchart. Articles were retrieved from PubMed and Embase databases (*n* = 521) and via citation tracking (*n* = 2). Articles published between the year of 1990 and the search date (May 2017; updated in January 2018) were included.

Retrieved articles were screened, duplicates were eliminated, and remaining citations were organized in EndNote (Clarivate Analytics, Philadelphia, PA) following Preferred Reporting Items for Systematic Reviews and Meta-Analyses (PRISMA) guidelines (Figure 1). First, two authors (EK and RT) created and agreed on a list of exclusion criteria, and independently screened a random sample of 66 titles and abstracts, blinded to authors and journal titles, for interrater reliability.^28^ The Cohen’s kappa reached 0.92, which is considered an almost perfect level of agreement.^29^ Second, the same two authors independently screened the total set of titles and abstracts, blinded to authors and journal titles.^30^ Data were compiled into an Excel workbook and consensus was reached on titles and abstracts that the authors evaluated in a different way.^31^ Articles were excluded (Figure 1) when e-cigarettes were not the research topic (*n* = 194). In addition, articles about toxicity, health, or health risks (*n* = 59); chemical–analytical research articles on liquid composition (*n* = 17); articles of which the title and abstract did not mention the word flavor or a specific flavor (*n* = 12); or review articles (*n* = 6) were excluded. In the third phase, the first author (EK) reviewed full-text articles to determine final eligibility. Articles were excluded if e-cigarettes were not the research topic (*n* = 11); the article described toxicology or health risks (*n* = 21) or chemical composition (*n* = 3); flavors were not the main research topic (*n* = 9); the article was a literature review (3); the topic was legislation (*n* = 3); the article was non-peer reviewed (*n* = 12); data were incomplete or insufficient (*n* = 5); or if the article did not use e-liquid flavor categories (*n* = 6). As we were interested in flavor classifications only to provide a broad overview of interpretations of researchers in order to develop a common flavor vocabulary, no articles were excluded based on quality (internal or external validity). Articles encountered via citation tracking that were considered eligible for inclusion were reviewed using the previously mentioned exclusion criteria (*n* = 2).

### Data Extraction and Synthesis

Included articles (*n* = 28) were analyzed by the first author using a data extraction table. The articles included have used a certain classification of e-cigarette flavors for data reduction, either to explain which flavors they used (eg, for experimental setups) or to categorize their results (eg, for surveys). For instance, Tackett et al.^6^ conducted a survey in which e-cigarette flavors were represented by six categories: fruity, bakery/dessert, tobacco blends, mint/menthol, candy/nuts, and coffee. From each article, the flavor categories used in the study design were extracted. A distinction was made between main flavor categories (eg, fruit or spice) and subcategories (specific e-liquid flavors that represent these categories, eg, lemon or cinnamon). For instance, the answer options of survey questions about consumers’ preferred e-liquid flavor (eg, “fruit” or “candy”) were main flavor categories, while the examples that researchers used to explain or specify these categories (eg, “e.g., cherry, watermelon, kiwi” or “e.g., bubble gum”) were considered specific e-liquid flavors that represent the main flavor categories. Another example: if researchers compared sweet flavors with nonsweet flavors, we considered “sweet” and “non-sweet” as the main flavor categories. The examples that researchers use as specification of these main categories were considered subcategories (eg, “chocolate” or “vanilla” as subcategory of sweet flavors, and “tobacco” or “menthol” as subcategory of nonsweet flavors).

Some of the main flavor categories or subcategories identified from literature were used in more than one article; hence, prevalence of each flavor category was determined. Results were summarized in Table 1 that shows each main flavor category and associated subcategories, (ie, flavor examples of the main categories), which were used in the articles reviewed.

**Table 1.** Main Flavor Categories Used in the Articles Reviewed (First Column), Prevalence Across Articles (Second Column), and the E-Liquid Flavors Mentioned as an Example of These Categories (Third Column). Main Categories Were Clustered on Similarity and Marked With a Color (Alternating Gray and White) to Distinguish Between Similar Categories. Individual E-Liquid Flavors in the Third Column Are Separated by a Comma. If an E-Liquid Flavor Was Mentioned as Example of a Particular Category in More Than One Article, Prevalence Is Indicated

Main flavor categories from literature (*n* = 43)	Prevalence	E-liquid flavors mentioned as example	References
Tobacco	19	Tobacco (*n* = 3), menthol	^4–6,11–13,27,34–37,39,41,42,44,47–50^
Tobacco or menthol	2	Tobacco, menthol	^43,46^
Menthol	10	Menthol (*n* = 2), mint (*n* = 2), menthol tobacco	^4,11,36,37,39,42,44,48,49,51^
Menthol/mint	7	Menthol, mint, peppermint	^5,6,12,27,34,35,51^
Mint	2		^11,40^
Nuts	3	Nuts (*n* = 2)	^5,39,44^
Nuts/spices	1	Almond, cinnamon, peanut butter, pecan	^27^
Seasonings	2	Cinnamon (*n* = 2), pepper (*n* = 2)	^39,44^
Spice	4	Cinnamon (*n* = 2), clove, nutmeg	^11,12,37,40^
Coffee	6	Cappuccino, espresso, latte	^4,6,11,35,37,41^
Coffee/alcohol	2		^12,40^
Coffee/tea	2	Cappuccino, coffee, espresso, tea	^27,34^
Alcohol	6	Absinthe, absolut, bourbon, champagne, (strawberry) daiquiri, mojitos, piña colada, rum, scotch	^11,27,34–37^
Beverages	3	Coffee (*n* = 3), alcoholic drinks, soda, tea (*n* = 2), wine (*n* = 2)	^39,44,45^
Beverages/drinks	1		^5^
Other beverages	1	Energy drinks, lemonades, sodas	^27^
Cherry	1		^4^
Fruit	18	Cherry (*n* = 7), strawberry (*n* = 7), apple (*n* = 4), blueberry (*n* = 4), mango (*n* =3), orange (*n* = 3), peach (*n* = 3), watermelon (*n* = 3), banana (*n* = 2), berry (*n* = 2), lemon (*n* = 2), pomegranate (*n* = 2), raspberry (*n* = 2), coconut, grape, green apple, lime, pear, plum	^5,6,11–13,27,34–45^
Bakery/dessert	1		^6^
Cream	2	Cake (*n* = 2), chocolate (*n* = 2), cookie (*n* = 2), custard (*n* = 2), milk (*n* = 2), vanilla (*n* = 2), butter, cheese, cream	^39,44^
Dessert	1	Chocolate	^38^
Dessert/sweets	1	Cakes, cereals, chocolate, donuts, ice cream, quick breads, vanilla, waffles	^27^
Food/dessert/spice	1	Banana foster, coffee, peaches, vanilla	^43^
Candy	7	Gummy bears (*n* = 3), licorice (*n* = 2), bubble gum, chocolate, Swedish fish, SweetTarts, vanilla	^27,33,34,36,37,40,46^
Candy or dessert	2	Chocolate (*n* = 2), apple pie, gummy bear, Jolly Rancher, vanilla	^11,12^
Candy/nuts	1	Almond, cotton candy, hazelnut, SweetTart	^6^
Caramel, vanilla, chocolate or cream	1		^34^
Chocolate	1	Chocolate	^42^
Chocolate/sweet	1		^35^
Sweet	7	Candy (*n* = 3), honey (*n* = 2), blackberry, candy floss, caramel, chocolate, cola, cotton candy, desserts, peach, sweet lemon tea, vanilla, watermelon	^3,5,39,41,44,45,48^
Vanilla	2		^11,35^
Flavorless	2		^3,35^
Unflavored	5	PG/VG base only (*n* = 2)	^12,27,38,42,51^
Combination of flavors	2	Blueberry champagne, bubble gum, tobacco, vanilla	^43,48^
Don’t know	2		^11,37^
Don’t know/other	1		^27^
Flavor	1	Buttery, chocolate, cinnamon, menthol	^54^
No flavor	1		^54^
Nonsweet	1	Menthol, mint, tobacco	^3^
Nontobacco	2	Cherry, peach, piña colada, vanilla	^47,50^
Other	7	Double espresso, pomegranate, vanilla bean	^5,11,35,37,40,46,48^
Other food	1	Cupcakes, muffins	^34^
Traditional flavors	1	Menthol, tobacco	^45^

PG = propylene glycol; VG = vegetable glycerine.

### Generation of the Flavor Wheel

The flavor categories extracted from literature served as a basis for our flavor wheel. Similar flavor categories were combined into one category. The name of this category was based on the name that was predominantly used in the articles reviewed (see prevalence numbers in Table 1). Resulting categories formed the inner layer of the flavor wheel.

The specific e-liquids that were used in literature as examples of the main categories were considered representative examples of the main categories. Therefore, each of the specific e-liquid flavors mentioned in literature was used as a subcategory in the outer layer of the flavor wheel. Brand names were excluded to solely include generic and generally known category names. Subcategories were sorted to be mutually exclusive; hence, each of the specific flavors used as example of a main category was associated to only one of the main categories. Classification of subcategories within main categories was based on classifications in articles reviewed (see Table 1), and flavor wheels from the food, alcohol, and fragrance industries.^19,20,22,24–26,32^

## Results

Database searches and citation tracking yielded 386 unique articles of which 25 met all inclusion criteria. A literature search update led to three additional eligible articles, resulting in a total inclusion of 28 publications. Most studies were conducted in the United States (*n* = 21). Other study locations were United Kingdom (*n* = 3), Canada (*n* = 2), Greece/Italy (*n* = 1), and China/United States (*n* = 1). An overview of study characteristics is added in Supplementary Table 2.

Analysis of flavor classifications used in the articles reviewed resulted in 43 unique main flavor categories, which are shown, including their prevalence across articles, in Table 1. Clustering similar categories resulted in 13 clusters of tobacco-, menthol-, fruit-, dessert-, alcohol-, nut-, spices-, candy-, coffee/tea-, beverages-, and sweet-like flavors, and unflavored e-liquids and unspecified flavors. The third column of Table 1 describes specific flavors mentioned as example of one of the main categories. For instance, Tackett et al.^6^ mentioned strawberry and blueberry as examples of their fruity category, and cotton candy, SweetTart, hazelnut, and almond as examples of the candy/nuts category. The prevalence of these specific flavors has been indicated if a flavor was mentioned as example of a particular category in more than one article.

The number of flavor categories used in the included articles varied from 1 to 11. For instance, Vasiljevic et al.^33^ conducted an experimental study with candy-flavored e-cigarettes only, whereas the survey of Yingst et al.^27^ distinguishes between 11 categories, being tobacco, menthol/mint, fruit, dessert/sweets, alcohol, nuts/spices, candy, coffee/tea, other beverages, unflavored, and don’t know/other flavors.

Considering flavor categories and classifications in literature, the overview of Table 1 shows that some of the flavor categories were used in more than one article. However, clustering similar categories shows that different category names were used to express the same types of flavors.

### Similarities in Flavor Classifications Across Literature

The category for alcohol-like flavors was named “alcohol” in each of the six articles using this category.^11,27,34–37^ Fruit-like flavors were classified as “fruit” in 18 articles^5,6,11–13,27,34–45^; only one of the articles reviewed used “cherry” as main category.^4^ Articles commonly used a separate “spice” category^11,12,37,40^; two articles used a “seasonings” category for flavors such as cinnamon and pepper.^39,44^ Regarding beverages, five articles used a category for “beverages,” “beverages/drinks,” or “other beverages.”^5,27,39,44,45^ Furthermore, “candy” was a common category name for candy-like flavors.^27,33,34,36,37,40,46^ Nineteen of the articles reviewed used a “tobacco” category for tobacco-like flavors.^4–6,11–13,27,34–37,39,41,42,44,47–50^ Finally, seven of the publications reviewed used an “unflavored”^12,27,38,42,51^ or “flavorless” category,^3,35^ explained by Litt et al.^42^ and Rosbrook and Green^51^ as a propylene glycol/vegetable glycerin base only. In conclusion, common categories used in literature are “alcohol,” “fruit,” “spice,” “beverages,” “candy,” “tobacco,” and “unflavored.”

### Differences in Flavor Classifications Across Literature

The differences in the naming of main flavor categories in literature are mostly related to menthol-, nuts-, coffee-, dessert- and sweet-like flavors, and to unspecified categories. Whereas “menthol” has been used as separate category in 10 studies,^4,11,36,37,39,42,44,48,49,51^ menthol has been used in combination with “mint”^5,6,12,27,34,35,51^ or “tobacco”^43,46^ as well. Even though menthol and tobacco are clearly different, researchers might have clustered these flavors because of the definition of characterizing flavors in cigarettes (flavors other than tobacco or menthol) by the US Food and Drug Administration,^52^ or by the fact that manufacturers commonly add menthol to tobacco products to increase sensory appeal.^9^ Clustering menthol with mint flavor might be related to fact that menthol is the major constituent of oils that are produced by *Mentha* plants, which have the well-known cooling minty taste and smell.^53^

Regarding nut flavors, three studies used a separate “nut” category,^5,39,44^ while others combined it with “spices”^27^ or “candy”.^6^ Similarly, six studies used a separate “coffee” category,^4,6,11,35,37,41^ while coffee has been classified together with “tea”^27,34^ or “alcohol”^12,40^ as well. “Dessert” has been mentioned as a separate category in Table 1,^38^ or together with “bakery”,^6^ “sweets”,^27^ “candy”,^11,12^ or “food/spice”.^43^ Dessert-like flavors were also classified as “cream.”^39,44^ Similarly, while “sweet” is a separate category in seven studies,^3,5,39,41,44,45,48^ some studies classified sweet flavors together with “dessert”^27^ or “chocolate”.^35^ In addition, flavors such as vanilla and chocolate have been used as main categories^11,34,35,42^ but were also part of the “sweet” category.^45^ Finally, the final rows of Table 1 represent 10 unspecified flavor categories such as “flavor,” “no flavor,” “non-tobacco,” “non-sweet,” “other,” “traditional flavors,” and “don’t know.”

Even though different names were used, the main categories described in this section could be summarized into “menthol”, “nuts”, “coffee”, “dessert”, “sweet”, and “other flavors”.

Besides differences in the naming of main categories, classification of specific e-liquid flavors within the main categories differed as well (third column of Table 1). Particularly e-liquids with a coffee, vanilla, and chocolate flavor were inconsistently classified: some articles classified these flavors within a different main category than others. Coffee-flavored e-liquids were classified within a separate category for “coffee/tea,”^27^ or within a “beverages,”^39,44,45^ or “food/dessert/spice” category.^43^ Vanilla-flavored e-liquids were classified within a broad range of categories, such as “candy or dessert,”^12^ “candy,”^36^ “food/dessert/spice,”^43^ “cream,”^39,44^ “sweet,”^45^ and “dessert/sweets.”^27^ Even though not consistently classified, vanilla seems a popular e-liquid flavor as it is mentioned as an example of three of the unspecified categories for other flavors as well.^43,46,47^ Similarly, besides being used as a separate category, chocolate-flavored e-liquids were classified within seven different flavor categories: “dessert,” “candy or dessert,” “candy,” “cream,” “sweet,” “desert/sweets,” and one of the unspecified categories.^11,12,27,36,38,39,42,44,45,54^

Thus, vanilla and chocolate were not classified exclusively to one category such as “dessert”, “candy”, or “beverages”. As vanilla and chocolate are often used as ingredients in sweet products, we consider these flavors general sweet flavors other than candy, dessert, or fruit.

### Proposed Flavor Wheel for E-Liquids

**Figure 2.**
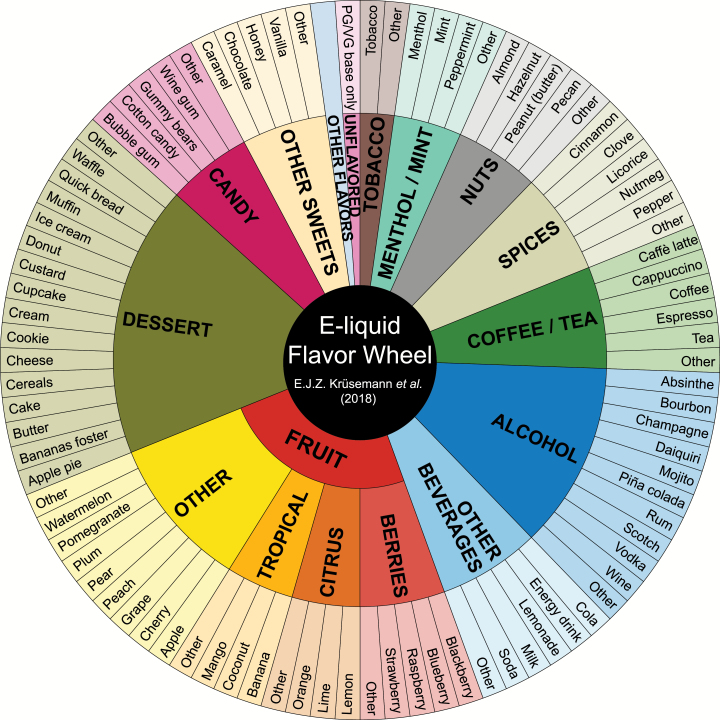
Proposed flavor wheel for classification of e-liquid flavors. The inner layer of the flavor wheel includes 13 main categories that were based on literature (first column, Table 1). The outer layer of the flavor wheel includes 90 subcategories that were extracted from the articles reviewed (third column, Table 1).

As a result of reviewing flavor classifications in literature, we propose a flavor wheel for e-liquids consisting of the following 13 main flavor categories: “tobacco,” “menthol/mint,” “nuts,” “spices,” “coffee/tea,” “alcohol,” “other beverages,” “fruit,” “dessert,” “candy,” “other sweets,” “other flavors,” and “unflavored.” Fruit flavors were divided into “berries,” “citrus,” “tropical,” and “other” fruits, similar to the division of the fruit category in the flavor wheels for wine, whiskey, coffee, and chocolate.^19,22,24,32^ The e-liquid flavor wheel is shown in Figure 2. The subcategories in the outer layer of the flavor wheel are represented by the specific e-liquid flavors that were used in literature as examples of main categories (third column, Table 1). As the categories from our flavor wheel are fully based on flavor classifications from reviewed articles, they do not by definition represent each e-liquid flavor available. Therefore, our flavor wheel contains a category for “other flavors” in order to classify flavors that have not been mentioned in literature.

## Discussion

We reviewed literature to determine which e-liquid flavors and flavor categories have been used in research. There was large variation in the naming of main flavor categories, and e-liquid flavors were not consistently classified within these categories. To classify the excessive number of e-liquid flavors in a consistent way, we propose a flavor wheel for e-liquids (Figure 2). Our flavor wheel includes 13 main categories (inner wheel) and 90 subcategories (outer wheel). The categories from our flavor wheel are fully based on flavor classifications in literature, from different countries.

### Guideline for Classifying E-Liquid Flavors Using Our Proposed Flavor Wheel

E-liquids are commonly classified based on marketing descriptions. Classifying e-cigarette flavors according to marketing involves brand names and flavor descriptions on packages or in advertisements. Flavor descriptions are used in promotion and marketing to create an association of the e-liquid’s flavor with a particular product that the consumer knows and preferably likes. Using these marketing descriptions for flavor classification requires common rules, as brand names regularly change and flavor descriptions are sensitive to interpretation differences. For instance, this review has shown that researchers have classified a particular flavor in different categories (eg, vanilla was classified as cream,^39,44^ candy,^36^ sweet,^45^ dessert/candy,^12^ or dessert/sweet^27^). Furthermore, e-liquids are not only marketed as single flavor such as strawberry or watermelon, but they can be associated with multiple flavor attributes. It could be questioned which of the flavor attributes should be used for classification, whether an e-liquid flavor can be associated with multiple categories, and how a distinction could be made between the “primary” flavor and “secondary” flavor attributes. For instance, of an e-liquid described as raspberry tea, is the primary flavor raspberry (eg, fruit) or tea? Similarly, if an e-liquid has multiple flavor attributes such as “a hint of tobacco, banana, rum and custard,” which of these attributes determines classification?

In order to minimize interpretation differences, to consistently classify e-liquids and distinguish primary from secondary flavors, we propose three steps as a guideline to classify e-liquid flavor using our flavor wheel:

#### Step 1

Distinguish primary from secondary e-liquid flavors. An e-liquid’s primary flavor is based on the flavor description that is associated with a particular product as a whole. If the e-liquid does not describe a clear product as a whole, the primary flavor is the first flavor attribute mentioned. If present, other flavor attributes are considered secondary flavors.

#### Step 2

Classify an e-liquid’s primary flavor in one of the 13 main categories as well as in one of the associated subcategories (inner wheel and outer wheel, respectively).

#### Step 3

Classify potential secondary flavors only in one of the subcategories (outer wheel).

The first step is based on the suggestion of Yingst et al.^27^ that flavors marketed as and meant to be associated with a particular product as a whole should be classified as a whole rather than the separate components of the e-liquid flavor. If an e-liquid’s brand name or flavor description cannot be associated with a product as a whole but the description contains a list of equal flavor descriptors instead, the first flavor attribute mentioned is considered the primary flavor; other flavor descriptors are secondary flavors. Thus, using previous examples, in e-liquids flavored as “raspberry tea” or “watermelon combined with kiwi and lemon,” we respectively consider tea and watermelon as primary flavors, whereas raspberry, kiwi, and lemon are secondary flavors. Furthermore, vanilla pudding and chocolate brownie are classified as “dessert”, whereas an e-liquid marketed purely as vanilla or chocolate flavor are classified as “other sweets.” Similarly, caramel candies such as toffee are classified as “candy”, whereas e-liquids simply marketed as caramel are classified as “other sweets.” Even though the flavor might be similar, we used marketing descriptions of the product as a whole for classification in order to minimize interpretation differences.

Our proposal is based on the rationale that a secondary flavor such as raspberry in raspberry tea should be included as well, because it distinguishes raspberry tea from other types of tea and thus is an important specification of the product. Extra flavors attributes besides the primary flavor are considered secondary flavors. The second and third steps suggest how to classify the primary and secondary flavors, respectively. The e-liquid flavor wheel contains 13 main categories (inner wheel) that are specified with 90 subcategories (outer wheel). According to the second step, the primary flavor should be classified in one of the main categories (inner wheel) and specified further in one of the associated subcategories (outer wheel), as the primary flavor is most important. According to the third step, secondary flavors, if present, should be classified only in one of the subcategories, as secondary flavors are solely meant for specification purposes. Examples of classifying primary as well as potential secondary flavors using marketing descriptions are provided in Table 2.

**Table 2.** Example of Classifying E-Liquids According to Their Primary and Secondary Flavors Using the Main and Subcategories of Our Proposed Flavor Wheel Shown in Figure 2. Classification Is Based on E-Liquid Marketing Descriptions

E-liquid	Flavor description	Main category primary flavor (inner wheel)	Subcategory primary flavor (outer wheel)	Secondary flavor? (yes/no)	Subcategories secondary flavor (outer wheel)
1	Raspberry tea	Coffee/tea	Tea	Yes	Raspberry
2	Watermelon combined with kiwi and lemon	Fruit	Watermelon	Yes	Kiwi, lemon
3	Strawberry with a hint of menthol	Fruit	Strawberry	Yes	Menthol
4	Chocolate	Other sweets	Chocolate	No	–
5	Bubble gum	Candy	Bubble gum	No	–
*etc.*					

Following the three steps when applying our flavor wheel allows classifying e-liquids in a way that most closely represents the flavor as a whole. The advantage of our flavor wheel over a linear list of flavor categories is that no hierarchy of flavor categories exists, and the flavor wheel distinguishes main categories in the inner wheel from subcategories in the outer wheel, and thus primary from, if present, secondary flavor attributes.

### Applications in Research

Our flavor wheel could be applied in multiple research disciplines. For instance, it might be a guideline in experimental study designs to select a representative sample of e-liquid flavors from different categories. In addition, e-liquid sales numbers could reveal information on popularity of particular flavors or flavor categories, and how demand of these e-liquid flavors persists over time. Using chemical–analytical research, flavor compositions of e-liquids could be compared. A large number of e-liquids could be measured using gas chromatography–mass spectrometry to investigate which flavor molecules are frequently present in e-liquids with particular flavors, and might thus be responsible for a particular flavor or flavor category from our flavor wheel. In sensory research on e-liquid flavors, the categories from the flavor wheel could be used as flavor attributes. E-liquids could be assessed by a panel of consumers or trained experts based on the intensity of particular flavor attributes to create a flavor profile. Flavor profiles created by panelists could be compared to e-liquid marketing descriptions to investigate to what extent consumers identify primary and potential secondary flavor attributes.

In addition, our flavor wheel can be used in research investigating liking and disliking of particular e-liquid flavors or flavor categories among different consumer groups such as smoking adults and nonsmoking youth. For instance, results of the studies included in this review on flavor liking show that e-cigarette users in general mostly prefer and/or use tobacco-flavored e-liquids and e-liquids with a sweet or fruit flavor.^3,5,6,12,27,34,35,37,39,43,44,47^ Males mostly seem to prefer tobacco-flavored e-liquids, whereas nontobacco (particularly sweet) flavors are more popular among females.^35,50^ Comparing adults with adolescents, sweet flavors are particularly popular among young e-cigarette users, while nonsweet flavors such as tobacco are more common among adults.^11,12,48^ Comparing smokers with nonsmokers, (adult) smokers are more interested in trying e-cigarettes with a tobacco or menthol flavor,^4,37,42,46,48^ whereas (younger) nonsmokers are more interested in trying fruit and sweet flavors.^4,36,41^ These conclusions show that sweet e-liquids are interesting for research on flavor liking. However, our review (Table 1) showed that “sweet”-flavored e-liquids have been inconsistently classified in literature, which may cause difficulties in data interpretation. Our flavor wheel provides a guideline to distinguish e-liquids with a dessert, candy, and beverage flavor from other sweet flavors such as vanilla or chocolate. Applying our flavor wheel in research on flavor liking will thus help to minimize interpretation differences and increase comparability of research results. Furthermore, our flavor wheel can be used to specify liking of main flavor categories into liking of specific e-liquid flavors (outer wheel) among different consumer groups.

Flavor liking in e-liquids could also be compared to liking and disliking of food products, as vaping and eating can both be considered forms of ingestive behavior (ie, the same route of administration [via nose and mouth] is followed, and the same type of psychological processes of perception and reward may be triggered). Flavors are important in both vaping and eating. For instance, children and adolescents have a high preference for sweet tastes and odors,^55^ which might explain why particularly sweet-, dessert-, and candy-flavored e-cigarettes are popular among youth.^3,4,12,17,36,41^ It would be interesting to further investigate similarities and differences between vaping and eating in relation to perception and reward.

In addition, our flavor wheel could be compared to flavor classifications in the food, alcohol, and fragrance industries, for instance to investigate if availability of e-liquid flavors is related to flavors that are commonly used in other products. A preliminary comparison between our e-liquid flavor wheel and the coffee, chocolate, wine, beer, whiskey, cigar, and fragrance wheels shows similarities and differences. For instance, each of the flavor wheels has a fruit category in their inner wheel.^19,20,22,24–26,32^ Similar to our wheel, categories for respectively nuts and spices are present in the inner wheels of the coffee-, cigar-, wine-, and chocolate-flavor wheel.^22,24,26,32^ Whereas tobacco is a main category in our flavor wheel, it is a subcategory of the brown fruit category of the chocolate wheel,^22^ the dried vegetative category of the wine aroma wheel,^32^ the plants category of the cigar flavor wheel,^26^ and the roasted category of the coffee flavor wheel.^24^ The candy, beverages, and dessert categories of our e-liquid wheel represent products as a whole, which are not recognized in other flavor wheels; except for the chocolate wheel, which includes subcategories such as cheesecake, butterscotch, toffee, candy “fruit tarts”, and a type of chocolate cake.^22^ The menthol/mint is a main category in our e-cigarette flavor wheel, whereas only the wine aroma wheel has a menthol subcategory.^32^ The main difference between the e-liquid and food flavor wheels is that our flavor wheel does not contain a floral category, while each of the other flavor wheels investigated has a floral category in their inner wheel.^19,20,22,24–26,32^ Strikingly, even though one article used “cheese” as part of their “cream” category,^39^ none of the articles reviewed used a main category for savory flavors, while research shows similar liking and reward for both sweet and savory food products.^56^ Because our flavor wheel is based on e-liquid flavors that have been used in research, it does not mean that no floral or savory flavored e-liquids exist. It would be interesting to investigate how many e-liquids with a floral or savory flavor are available, and how liking of these e-liquids relates to liking of savory and floral-flavored food products.

Our flavor wheel may be also be used for development and analysis of survey items. For instance, researchers could use the main and/or subcategories of the flavor wheel as answer options for multiple choice questions related to e-liquid flavor use and/or preferences. The flavor categories could also be used to (manually) classify open-ended responses from consumers to similar survey questions. In this way, the flavor wheel facilitates communication between researchers and real-world users, which helps to understand consumer liking and disliking of certain e-liquid flavors.

### Applications in Policy

Consistent classification of e-liquid flavors by consumers as well as researchers will improve data accuracy, minimize interpretation differences, and increase comparability of research results across studies. Research results could be used by policy makers for regulation of particular e-liquid flavors or flavor categories from the flavor wheel. The classification rules from Yingst et al.^27^ were based on the possibility that the same regulations for flavors in cigarettes would be applied to e-liquids. In cigarettes, characterizing flavors have been prohibited, which are defined by the European Union as “flavors other than the one of tobacco” and by the US Food and Drug Administration as “flavors other than tobacco or menthol.”^52,57^ Therefore, Yingst et al.^27^ aimed to distinguish e-liquids with an exclusive tobacco flavor from e-liquids also having other flavor attributes. According to their classification rules, e-liquids marketed as “pipe tobacco with a hint of cherry” would be classified as fruit.

However, as all e-liquids have a flavor, it might be difficult to compare e-liquids with cigarettes from a regulation point of view. Furthermore, considering the product as a whole and the first flavor mentioned, the primary flavor attribute of the example according to our flavor wheel would be tobacco. According to our proposal, this e-liquid would be classified in the main “tobacco” category (inner wheel) with “pipe tobacco” as subcategory (outer wheel), with an additional secondary flavor in a “cherry” subcategory (outer wheel). Our flavor wheel thus allows to distinguish e-liquids with a primary tobacco flavor from e-liquids marketed as having a primary tobacco flavor and additional secondary flavors other than tobacco. In this way, each of the flavor attributes that are used for marketing of e-liquids could be considered for regulation of e-liquid flavors.

Furthermore, (characterizing) flavors in tobacco cigarettes are prohibited because they increase attractiveness and thereby facilitate smoking initiation among young people.^58,59^ Flavors in e-cigarettes are not only attractive to young people, but are also associated with higher rates of smoking cessation among adults.^6^ Sensory research using our flavor wheel will provide more insight in liking of e-liquid flavors and/or flavor categories among these different consumer groups. Policy makers could use research results to regulate e-liquid flavors in a way that e-cigarettes are attractive to adult smokers and unattractive to young nonsmokers.

### Future Research

The categories from our flavor wheel should be corroborated to determine whether the wheel is complete or additional categories are required. For instance, as categories from our flavor wheel were mainly based on studies performed in the United States, research on e-liquid flavors offered by retail Web sites from different countries might identify new or other flavors that are not covered by our flavor wheel. Preliminary market observations have revealed the availability of e-liquid flavors that have not been used in the study design of the articles reviewed, such as rose and chicken. E-liquids flavored as such would be classified in the “other flavor” category of our flavor wheel. Similar to the need for modification of the wine aroma wheel,^32^ future research might reveal the need to specify the category for “other flavors” into additional categories such as “floral” or “savory”. Future research should also investigate if our flavor wheel is complete and not open to misinterpretation by having a panel of consumers classify a large sample of e-liquid flavor descriptions on the basis of the proposed flavor wheel. Statistical data on e-liquid classification by the panel will show if panelists follow the classification steps and apply the flavor wheel in a consistent, repeatable, and reproducible way.

## Conclusions

A large variation in the naming of flavor categories was found in literature, and e-liquid flavors were not consistently classified. We propose an e-liquid flavor wheel including three steps for systematic classification of e-liquids based on their marketing descriptions. The flavor wheel includes 13 main categories (inner wheel) and 90 subcategories (outer wheel) that aim to create a shared flavor vocabulary for a broad range of potential users. Applying the flavor wheel in research will minimize interpretation differences, increase comparability of research results, and support policy makers in developing rules for regulation of e-liquid flavors.

## Supplementary Material

Supplementary information can be found online at http://www.ntr.oxfordjournals.org.

## Funding

This work was supported by the Dutch Ministry of Health, Welfare and Sport (project number 5.7.1).

## Declaration of Interests

*None declared*.

## Supplementary Material

nty101_suppl_Supplementary_Information
