# Supplementary material for: An E-Liquid Flavor Wheel: A Shared Vocabulary Based on Systematically Reviewing E-Liquid Flavor Classifications in Literature
Source: Nicotine Tob Res. 2018 May 18;21(10):1310–9. doi: 10.1093/ntr/nty101 (PMC6751518; doi:10.1093/ntr/nty101)
Supplement: nty101_suppl_Supplementary_Information [file nty101_suppl_supplementary_information.pdf]

Supplementary information

An E-Liquid Flavor Wheel: A Shared Vocabulary Based on Systematically Reviewing E-Liquid Flavor Classifications in Literature

Erna JZ Krüsemann, MSc[1,2], Sanne Boesveldt, PhD[2], Kees de Graaf, PhD[2], Reinskje Talhout, PhD[1]

[1]National Institute for Public Health and the Environment (RIVM), Centre for Health Protection, Antonie van Leeuwenhoeklaan 9, 3721 MA Bilthoven, The Netherlands; [2]Division of Human Nutrition, Wageningen University, PO Box 17, 6700 AA Wageningen, The Netherlands

Corresponding Author: Erna JZ Krüsemann, MSc, National Institute for Public Health and the Environment (RIVM), Centre for Health Protection, Antonie van Leeuwenhoeklaan 9, 3721 MA Bilthoven, The Netherlands; E-mail: erna.krusemann@rivm.nl

Table of Contents

Supplementary Table 1: PubMed database search strategy........S2

Supplementary Table 2: Characteristics of the included publications (n=28)........S3

References........S7

Supplementary Table 1: PubMed database search strategy

Search	Query	Items found
**#19**	Search **(#8 or #11 or #13 or #14 or #16 or #17)**<br>Filters: **Publication date from 1990/01/01 to 2017/05/17**	197
**#18**	Search **(#8 or #11 or #13 or #14 or #16 or #17)**	214
**#17**	Search **((#4 or #9) and #15)**	4
**#16**	Search **((#5 or #10) and #15)**	140
**#15**	Search **(learning*[Title] or wanting*[Title] or liking*[Title])**	74125
**#14**	Search **(#9 and #12)**	13
**#13**	Search **(#4 and #12)**	14
**#12**	Search **"perception"[MeSH Major Topic]**	214642
**#11**	Search **(#9 and #10)**	21
**#10**	Search **"flavoring agents"[MeSH Major Topic]**	7989
**#9**	Search **electronic cigarette[MeSH Major Topic]**	1014
**#8**	Search **(#4 and #7)**	47
**#7**	Search **(#5 or #6)**	4049
**#6**	Search **(consumer*[Title] and preference*[Title])**	261
**#5**	Search **(flavour*[Title] or flavor*[Title])**	3797
**#4**	Search **(#1 or #2 or #3)**	1855
**#3**	Search **electronic[Title] and nicotine[Title]**	205
**#2**	Search **electronic cigar*[Title]**	900
**#1**	Search **e-cigar*[Title]**	889

Supplementary Table 2: Characteristics of the included publications (n=28)

Authors, year (country)	Study population, sample size and mean age (SD)	Study design	Flavor classification
**Audrain-McGovern *et al*, 2016 (USA) [1]**	Young adult smokers, n=32, mean age 25.0 (3.0)	Experimental laboratory session	Unflavored<br>Fruit *(green apple)*<br>Dessert *(chocolate)*
**Berg, 2016 (USA) [2]**	Adults aged 18-34 years living in US, n=1567, mean age 25.2 (5.1)	Cross-sectional survey	Fruit<br>Caramel, vanilla, chocolate or cream<br>Candy *(e.g., licorice, gummy bears)*<br>Menthol/mint<br>Tobacco<br>Coffee/tea<br>Alcohol *(e.g., mojitos, daiquiris)*<br>Other food *(e.g., cupcakes, muffins)*
**Chen and Zeng, 2017 (USA)* [3]**	14,433 e-liquid reviews from the JuiceDB website between June 2013 and November 2015	Longitudinal content analysis	Fruit *(strawberry, banana, apple, blueberry, mango, cherry, orange, lemon, waterlemon, raspberry, pomegranate, pear, plum, grape, lime)*<br>Cream *(cream, vanilla, custard, milk, chocolate, cake, cookie, cheese, butter)*<br>Tobacco *(tobacco)*<br>Menthol *(menthol, mint)*<br>Beverages *(coffee, tea, wine)*<br>Sweet *(candy, honey, caramel)*<br>Seasonings *(cinnamon, pepper)*<br>Nuts *(nuts)*
**Chu *et al*, 2015 (USA) [4]**	6 months of tweets from 2 e-cigarette brands (Blu owned by Lorillard and V2 owned by VMR), n=1180	Longitudinal content analysis	Flavor *(e.g., buttery, menthol, chocolate, cinnamon, and so on - excluding tobacco)*<br>No flavor
**Cooper *et al*, 2016 (USA) [5]**	A probability-design sample of public schools. Data from a rapid response surveillance system (TATAMS) with 6th, 8th and 10th grade students (N=434,601, n=3704)	Cross-sectional survey	Mint<br>Candy<br>Fruit<br>Coffee/alcohol<br>Spice<br>Other
**Czoli *et al*, 2016 (Canada) [6]**	Non-smoking youth and young adults aged 16–24 years, mean age 20.6 (2.8); smoking youth and young adults aged 16–24 years, mean age 21.4 (2.1); and smoking adults aged 25 years and older, mean age 49.0 (12.2). Recruited through GMI, n=915.	Experimental online assessment	Tobacco<br>Menthol<br>Coffee<br>Cherry
**Dawkins *et al*, 2013 (UK) [7]**	A restricted sample of TECC and Totally Wicked E-Liquid (TWEL) users (the two most widely-used brands in the UK) recruited via their websites, n=1347, mean age 43.39 (11.99)	Cross-sectional survey	Tobacco<br>Fruit<br>Menthol/mint<br>Chocolate/sweet<br>Coffee<br>Other<br>Vanilla<br>Alcohol<br>Flavorless
**Farsalinos *et al*, 2013 (Greece / Italy) [8]**	Dedicated adult e-cigarette users of any age, n=4618, median age 40 (32–49 interquartile range).	Cross-sectional survey	Tobacco<br>Menthol/mint<br>Sweet<br>Nuts

			Fruit<br>Beverages/drinks<br>Other
**Ford *et al*, 2016 (UK) [9]**	Data from the 2014 Youth Tobacco Policy Survey among 11-16 year olds across the UK, n=1205, mean age 13.5	Cross-sectional survey	Tobacco<br>Fruit *(cherry)*<br>Sweet *(candy floss)*<br>Coffee
**Goldenson *et al*, 2016 (USA) [10]**	Young adult vapers aged 19-34 years, n=20, mean age 26.3 ± 4.6	Experimental laboratory session	Sweet *(peach,watermelon, blackberry, cotton candy, cola and sweet lemon tea)*<br>Non-sweet *(mint, tobacco and menthol)*<br>Flavorless
**Harrell *et al*, 2017 (USA) [11]**	(A) Youth aged 12–17 years recruited from TATAMS (N=461,069, n=3907);<br>(B) Young adults aged 18–29 years recruited from M-PACT (N=13,714, n=5482);<br>(C) Older adults aged 30+ years recruited from TPRPS (N=8135, n=6015)	Cross-sectional survey	Tobacco<br>Menthol/mint<br>Fruit *(e.g., cherry, strawberry)*<br>Candy or dessert *(e.g., respectively gummy bear or chocolate/vanilla)*<br>Coffee/alcohol<br>Spice *(e.g., cinnamon)*<br>Unflavored
**Kim *et al*, 2016 (USA) [12]**	E-cigarette sole or dual users, n=31, mean age 33.6±10.9	Experimental laboratory session	Tobacco *(tobacco and menthol: resp. Classic Tobacco and Magnificent Menthol)*<br>Non-tobacco *(cherry, piña colada, peach, vanilla: resp. Cherry Crush, Piña Colada, Peach Schnapps, and Vivid Vanilla)*
**Krishnan-Sarin *et al*, 2015 (USA) [13]**	(A) High school students from Connecticut, n=3614, mean age 15.63 (1.20)<br>(B) Middle school students from Connecticut, n=1166, mean age 12.18 (0.90)	Cross-sectional survey	Menthol<br>Tobacco<br>Sweet<br>Combination of flavors<br>Other
**Litt *et al*, 2016 (USA) [14]**	Current cigarette smokers aged 18-55 years, n=88, mean age 36.3 (10.3)	Experimental laboratory session and field study	Unflavored *(PG/VG base only)*<br>Tobacco<br>Menthol<br>Fruit *(cherry)*<br>Chocolate *(chocolate)*
**Morean *et al*, 2018 (USA)* [15]**	Adolescent past-month e-cigarette users from 5 high schools, n=396, mean age 16.18 (1.18); adult past month-e-cigarette users, n=590, mean age 34.25 (9.89)	Cross-sectional survey	Tobacco<br>Menthol<br>Mint<br>Fruit *(e.g., strawberry, blueberry, or peach)*<br>Vanilla<br>Candy/dessert *(e.g., apple pie, chocolate, or Jolly Rancher)*<br>Spice *(e.g., clove, cinnamon or nutmeg)*<br>Alcohol *(e.g., piña colada, strawberry daiquiri, or bourbon)*<br>Coffee *(e.g., espresso, latte, or cappuccino)*<br>Other<br>Don't know
**Oncken *et al*, 2015 (USA) [16]**	Non-treatment seeking smokers who were willing to try e-cigarettes for 2 weeks and abstain from cigarette smoking, n=20, mean age 42.2 (9.7)	Experimental field study and laboratory session, and cross-sectional survey	Menthol *(menthol tobacco)*<br>Tobacco
**Pepper *et al*, 2016 (USA) [17]**	A national probability sample of USA adolescents aged 13–17 years, n=1125, mean age 15.1 (1.4)	Experimental survey	Tobacco<br>Alcohol *(e.g., scotch or champagne)*<br>Menthol<br>Candy *(e.g., chocolate or vanilla)*<br>Fruit *(e.g., cherry or peach)*

**Piñeiro *et al*, 2015 (USA) [18]**	E-cig users aged 18-29, 30-44, 45-59, and ≥60 years, n=1815	Cross-sectional survey	Tobacco<br>Non-tobacco
**Rosbrook and Green, 2016 (USA) [19]**	Adult daily smokers aged 18-45 years, n=32	Experimental laboratory session	Menthol<br>Menthol/mint<br>Unflavored *(PG/VG base only)*
**Shiffman *et al*, 2015 (USA) [20]**	Non-smoking teens aged 13-17 years, mean age 15.9 (1.1); adult smokers aged 19-80 years, mean age 43.7 (14.5); n=648	Experimental online assessment	Tobacco or menthol *(tobacco, menthol: Classic Tobacco, Menthol, Dark Tobacco Blend)*<br>Candy *(bubble gum and gummy bear: Bubble Gum, Cotton Candy, Gummy Bear)*<br>Other *(e.g., pomegrenate, vanilla bean, double espresso: Black & Blue Berry, Blood Orange, Butter Crunch, Double Espresso, Peach Tea, Pomegrenate, Raspberry, Single Malt Scotch, Vanilla Bean)*
**Shiplo *et al*, 2015 (Canada) [21]**	Younger non-smokers (mean age 20.51), younger smokers (21.35), and older smokers (48.52). Recruited through GMI, n=1095	Cross-sectional survey	Fruit<br>Menthol<br>Tobacco<br>Candy<br>Coffee<br>Spice<br>Alcohol<br>Other<br>Don't know
**Soule *et al*, 2016 (USA) [22]**	Adult experienced e-cig users, n=46, mean age 38.5 (10.52)	Mixed-Method: online assessment and survey	Food/Dessert/Spice *(e.g., vanilla, banana foster, peaches, coffee)*<br>Fruit *(e.g., watermelon, mango)*<br>Tobacco or menthol<br>Combination of flavors *(e.g., bubble gum, blueberry champagne, vanilla and tobacco)*
**St.Helen *et al*, 2017 (USA)* [23]**	Exclusive e-cigarette users or dual users (<5 cig/day), n=14, mean age 32.3 (13.8)	Experimental laboratory session	Fruit *(strawberry)*<br>Tobacco *(tobacco)*
**Tackett *et al*, 2015 (USA) [24]**	Adult vape store customers at four retail locations in the Midwestern United States, n=215, mean age 36.2 (13.0)	Cross-sectional survey	Fruit *(e.g., strawberry, blueberry)*<br>Bakery/dessert<br>Tobacco<br>Menthol/mint<br>Candy/nuts *(e.g., cotton candy, SweetTart, Hazelnut, Almond)*<br>Coffee
**Vasiljevic *et al*, 2015 (UK) [25]**	English school children aged 11–16 years, n=598, mean age 13.16 (1.46)	Experimental exposure assessment	Candy
**Wang *et al*, 2015 (China / USA) [26]**	27,638 flavor-related posts and 7,376 brand-related posts in 10 subreddit communities	Longitudinal content analysis	Fruit *(strawberry, banana, apple, peach, blueberry, mango, cherry, orange, lemon, watermelon, raspberry, pomegranate)*<br>Cream *(vanilla, custard, milk, chocolate, cake, cookie)*<br>Tobacco<br>Menthol *(menthol, mint)*<br>Beverages *(coffee, tea, wine)*<br>Sweet *(candy, honey)*<br>Seasonings *(cinnamon, pepper)*<br>Nuts *(nuts)*
**Yingst *et al*, 2015 (USA) [27]**	E-cig users, n=4421, mean age 40.1 (12.7)	Cross-sectional survey	Traditional flavors *(tobacco or menthol)*<br>Fruit *(e.g., cherry, berry, apple)*<br>Sweet *(e.g., chocolate, vanilla, desserts, candies)*<br>Beverages *(e.g., coffee, alcoholic drinks, soda)*
**Yingst *et al*, 2017 (USA) [28]**	Current e-cig users, who were either current or former smokers, at least 18 years of	Cross-sectional survey	Tobacco<br>Menthol/mint *(menthol, mint, peppermint)*<br>Fruit *(e.g., apple, strawberry, coconut, orange,*

age, n=3716, mean age 40.4	*berries)*<br>Dessert/sweets *(e.g., chocolate, vanilla, quick breads, cakes, waffles, donuts, cereals, and ice cream)*<br>Alcohol *(e.g., rum, absinthe, absolut)*<br>Nuts/spices *(e.g., peanut butter, almond, cinnamon, pecan)*<br>Candy *(e.g., licorice, sweetTARTS, gummy bears, Swedish fish)*<br>Coffee/tea *(e.g., coffee, tea, espresso, cappuccino)*<br>Other beverages *(e.g., sodas, energy drinks, lemonades)*<br>Unflavored<br>Don't know/other

*Articles included as result of the literature search update (n=3)

References

1. Audrain-McGovern, J., A.A. Strasser, and E.P. Wileyto, *The impact of flavoring on the rewarding and reinforcing value of e-cigarettes with nicotine among young adult smokers*. Drug and alcohol dependence, 2016. **166**: p. 263-7.
2. Berg, C.J., *Preferred flavors and reasons for e-cigarette use and discontinued use among never, current, and former smokers*. International journal of public health, 2016. **61**(2): p. 225-236.
3. Chen, Z. and D.D. Zeng, *Mining online e-liquid reviews for opinion polarities about e-liquid features*. BMC Public Health, 2017. **17**(1): p. 633.
4. Chu, K.H., et al., *Electronic Cigarettes on Twitter - Spreading the Appeal of Flavors*. Tobacco regulatory science, 2015. **1**(1): p. 36-41.
5. Cooper, M., et al., *Flavorings and Perceived Harm and Addictiveness of E-cigarettes among Youth*. Tobacco regulatory science, 2016. **2**(3): p. 278-289.
6. Czoli, C.D., et al., *Consumer preferences for electronic cigarettes: results from a discrete choice experiment*. Tobacco control, 2016. **25**(e1): p. e30-6.
7. Dawkins, L., et al., *'Vaping' profiles and preferences: an online survey of electronic cigarette users*. Addiction, 2013. **108**(6): p. 1115-25.
8. Farsalinos, K.E., et al., *Impact of flavour variability on electronic cigarette use experience: An internet survey*. International Journal of Environmental Research and Public Health, 2013. **10**(12): p. 7272-7282.
9. Ford, A., et al., *Adolescents' responses to the promotion and flavouring of e-cigarettes*. International journal of public health, 2016. **61**(2): p. 215-24.
10. Goldenson, N.I., et al., *Effects of sweet flavorings and nicotine on the appeal and sensory properties of e-cigarettes among young adult vapers: Application of a novel methodology*. Drug and Alcohol Dependence, 2016. **168**: p. 176-180.
11. Harrell, M.B., et al., *Flavored e-cigarette use: Characterizing youth, young adult, and adult users*. Preventive Medicine Reports, 2017. **5**: p. 33-40.
12. Kim, H., et al., *Role of sweet and other flavours in liking and disliking of electronic cigarettes*. Tobacco control, 2016. **25**(Suppl 2): p. ii55-ii61.
13. Krishnan-Sarin, S., et al., *E-cigarette use among high school and middle school adolescents in Connecticut*. Nicotine and Tobacco Research, 2015. **17**(7): p. 810-818.
14. Litt, M.D., V. Duffy, and C. Oncken, *Cigarette smoking and electronic cigarette vaping patterns as a function of e-cigarette flavourings*. Tobacco control, 2016. **25**(Suppl 2): p. ii67-ii72.
15. Morean, M.E., et al., *Preferring more e-cigarette flavors is associated with e-cigarette use frequency among adolescents but not adults*. PLoS One, 2018. **13**(1): p. e0189015.
16. Oncken, C.A., et al., *Nicotine concentrations with electronic cigarette use: Effects of sex and flavor*. Nicotine and Tobacco Research, 2014. **17**(4): p. 473-478.
17. Pepper, J.K., K.M. Ribisl, and N.T. Brewer, *Adolescents' interest in trying flavoured e-cigarettes*. Tobacco control, 2016. **25**(Suppl 2): p. ii62-ii66.
18. Piñeiro, B., et al., *Gender differences in use and expectancies of e-cigarettes: Online survey results*. Addictive Behaviors, 2016. **52**: p. 91-97.
19. Rosbrook, K. and B.G. Green, *Sensory effects of Menthol and Nicotine in an E-cigarette*. Nicotine and Tobacco Research, 2016. **18**(7): p. 1588-1595.
20. Shiffman, S., et al., *The impact of flavor descriptors on nonsmoking teens' and adult smokers' interest in electronic cigarettes*. Nicotine and Tobacco Research, 2015. **17**(10): p. 1255-1262.

21. Shiplo, S., C.D. Czoli, and D. Hammond, *E-cigarette use in Canada: Prevalence and patterns of use in a regulated market.* BMJ Open, 2015. **5**(8).
22. Soule, E.K., et al., *Reasons for using flavored liquids among electronic cigarette users: A concept mapping study*. Drug and Alcohol Dependence, 2016. **166**: p. 168-176.
23. St Helen, G., et al., *Impact of e-liquid flavors on nicotine intake and pharmacology of e-cigarettes*. Drug Alcohol Depend, 2017. **178**: p. 391-398.
24. Tackett, A.P., et al., *Biochemically verified smoking cessation and vaping beliefs among vape store customers*. Addiction, 2015. **110**(5): p. 868-74.
25. Vasiljevic, M., D.C. Petrescu, and T.M. Marteau, *Impact of advertisements promoting candy-like flavoured e-cigarettes on appeal of tobacco smoking among children: an experimental study.* Tobacco control, 2016. **25**(e2): p. e107-e112.
26. Wang, L., et al., *An examination of electronic cigarette content on social media: Analysis of e-cigarette flavor content on reddit.* International Journal of Environmental Research and Public Health, 2015. **12**(11): p. 14933-14952.
27. Yingst, J.M., et al., *Factors associated with electronic cigarette users' device preferences and transition from first generation to advanced generation devices.* Nicotine and Tobacco Research, 2015. **17**(10): p. 1242-1246.
28. Yingst, J.M., et al., *A method for classifying user-reported electronic cigarette liquid flavors*. Nicotine & tobacco research : official journal of the Society for Research on Nicotine and Tobacco, 2017.
